# CD105 (Endoglin) as negative prognostic factor in AML

**DOI:** 10.1038/s41598-019-54767-x

**Published:** 2019-12-04

**Authors:** Joseph Kauer, Karolin Schwartz, Claudia Tandler, Clemens Hinterleitner, Malte Roerden, Gundram Jung, Helmut R. Salih, Jonas S. Heitmann, Melanie Märklin

**Affiliations:** 1 Institute for Cell Biology, Department of Immunology, University of Tübingen, Tübingen, Germany; 2 German Cancer Consortium (DKTK), DKFZ partner site Tübingen, Tübingen, Germany; 3 Clinical Collaboration Unit Translational Immunology, German Cancer Consortium (DKTK), Department of Internal Medicine, University Hospital Tübingen, Tübingen, Germany; 4 DFG Cluster of Excellence 2180 ‘Image-guided and Functional Instructed Tumor Therapy’ (IFIT), University of Tübingen, Tübingen, Germany; 5 University Hospital Tübingen, Dept. of Oncology, Haematology and Immunology, Tübingen, Germany

**Keywords:** Immunology, Acute myeloid leukaemia

## Abstract

While several genetic and morphological markers are established and serve to guide therapy of acute myeloid leukaemia (AML), there is still profound need to identify additional markers to better stratify patients. CD105 (Endoglin) is a type I transmembrane protein reported to induce activation and proliferation of endothelial cells. In addition, CD105 is expressed in haematological malignancies and the vessels of solid tumours. Here, CD105 associates with unfavourable disease course, but so far no data are available on the prognostic relevance of CD105 in haematological malignancies. We here generated a novel CD105 antibody for analysis of expression and prognostic relevance of CD105 in a cohort of 62 AML patients. Flow cytometric analysis revealed substantial expression in the various AML FAB types, with FAB M3 type displaying significantly lower surface levels. Next we established a cut-off specific fluorescence level of 5.22 using receiver-operating characteristics, which allowed to group patients in cases with CD105^lo^ and CD105^hi^ surface expression and revealed that high CD105 expression correlated significantly with poor overall and progression free survival. In conclusion, we here identify CD105 expression as a novel prognostic marker in AML, which may serve to optimize follow up and treatment decisions for AML patients.

## Introduction

Acute myeloid leukaemia (AML) is a malignant disorder of the haematopoietic system with a high mortality rate and variable prognosis^1^. Commonly used prognostic factors are cytogenetic and molecular features including inv(16), t(8;21), t(15;17) as well as IDH and FLT3 mutations^2–4^. In addition, immunophenotyping by flow cytometry provides valuable prognostic information^5^. However, there is still room for improvement of risk assessment, and thus identification of novel biomarkers for the prediction of remission rates, risk of relapse and overall outcome holds promise to improve the treatment of AML by guiding treatment decisions^6^.

CD105 (Endoglin) is a type I transmembrane protein which serves as an auxiliary receptor within the transforming growth factor (TGF) beta signalling complex and induces activation and proliferation of endothelial cells^7,8^. CD105 is highly expressed on vascular endothelium^9^ and could be detected on stromal cells, melanocytes and different healthy cells of the haematopoietic system^10–12^. Moreover, CD105 is specifically upregulated on proliferating endothelium and was found to be expressed on tumour vessels of brain, breast, colon, lung and stomach cancer^13,14^. The expression of CD105 on tumour vessels reportedly correlates with poor prognosis in endometrial, colorectal, breast, prostate, and non small cell lung cancer^15–20^. CD105 expression has also been described in myelodysplastic syndrome (MDS), acute lymphoblastic leukaemia (ALL) and AML^21–23^, but its prognostic relevance in these and other haematopoietic malignancies has not been assessed so far. We here report on a newly generated CD105 antibody that was used to analyse CD105 expression in a cohort of 62 AML patients and identified a strong correlation of high CD105 expression with adverse disease outcome.

## Results

### Generation of a novel CD105 antibody

**Figure 1:**
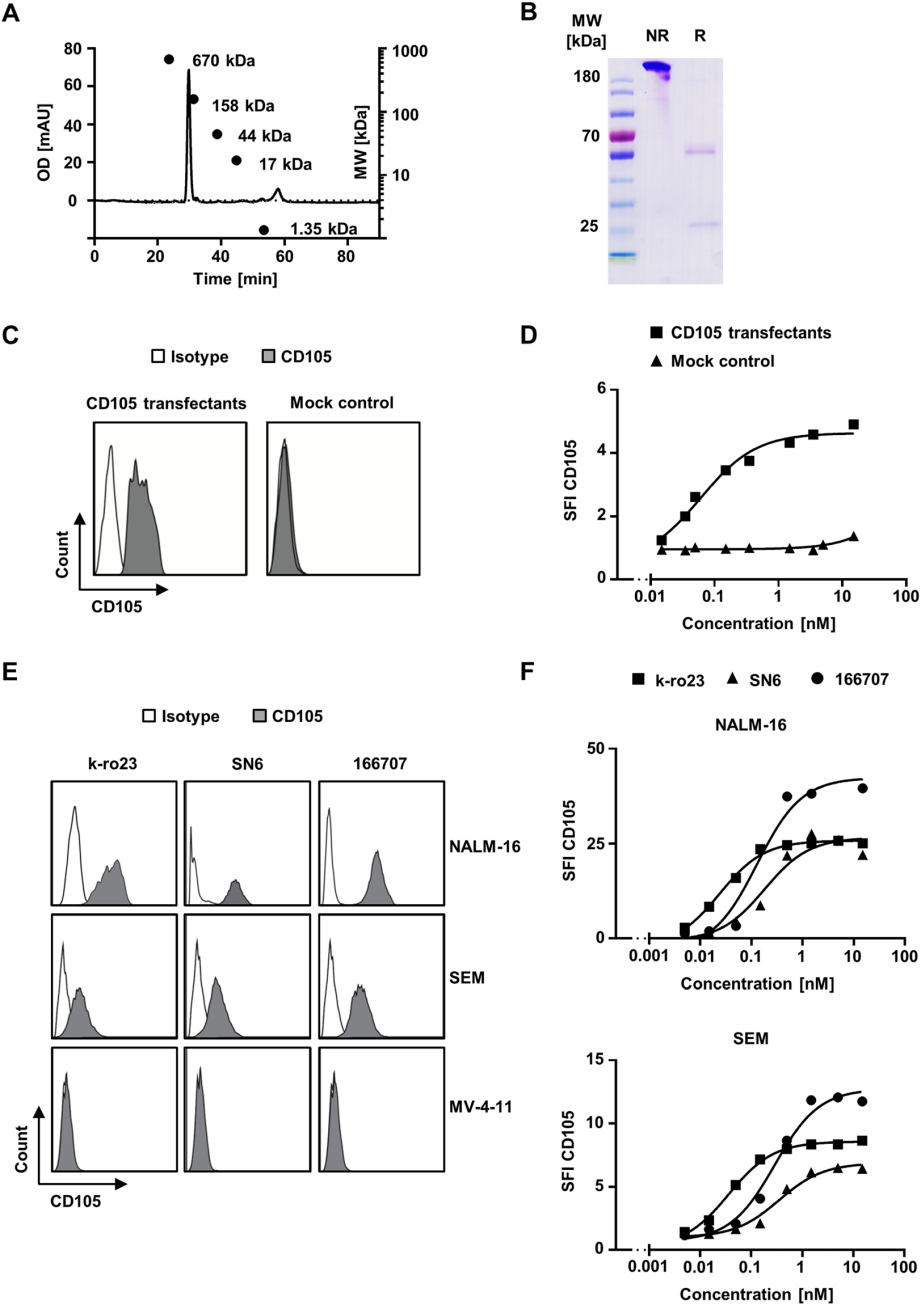
Generation and functional characterization of CD105 antibody K-ro23. The CD105 antibody K-ro23 was generated as described in the methods section. (**A**) Size exclusion chromatography after the initial purification with protein A affinity chromatography. (**B**) SDS-PAGE analysis under reducing (R) and non-reducing conditions (NR). (**C**) Flow cytometry based detection of specific binding of the CD105 antibody (shaded peaks) and an isotype control (open peaks) at 1 nM on CD105 transfected Sp2/0 and mock transfected Sp2/0, and (**D**) analysis of the binding capacity of the CD105 antibody on CD105 transfected and mock transfected Sp2/0 as negative control. (**E**) Comparison of the specific binding of K-ro23 and the commercial clones SN6 and 166707 (shaded peaks) compared to the respective isotype controls (open peaks) at 5 nM on acute leukaemia cell lines. (**F**) Titration of the different CD105 clones on acute leukaemia cell lines. Specific fluorescence intensity (SFI) levels are shown.

The murine CD105 IgG2b antibody K-ro23 was generated as described in Methods section. SDS-PAGE analyses and size exclusion chromatography confirmed the presence of an intact IgG molecule with the respective light and heavy chains as well as the absence of aggregates as depicted in Fig. 1A,B. Detection of K-ro23 bound to CD105-transfected Sp2/0 as compared to mock controls by flow cytometry confirmed the specific binding of the antibody, and saturating concentrations were achieved with <1 nM (Fig. 1C,D). Binding analyses with two commercially available antibodies (SN6, 166707) on different acute leukaemic cell lines as well as on different solid tumour cell lines confirmed specific binding and the absence of false-positive signals (Figs. 1E and S1A). Titration experiments showed a higher affinity of K-ro23 to CD105 as reflected by saturation at lower concentrations and compared to SN6 similar signals at high concentrations, which were both slightly lower than 166707 (Figs. 1F and S1B).

### Clinical characteristics of the AML patient cohort

For analysis of CD105 expression we employed primary AML samples of 62 patients. The clinical characteristics of the patients are given in Table 1. Nineteen patients presented with undifferentiated leukaemia (M0: n = 4, M1: n = 15), 20 with immature granulocytic leukaemia (M2: n = 16, M3: n = 4) and 14 with monocytic leukaemia (M4: n = 8, M5: n = 6); nine patients had erythroleukaemia (M6). In 46 patients, primary and in 16 patients secondary AML was diagnosed. The age ranged from 21–86 years (with a median of 64 years) with a male:female ratio of 1:1.07. Patients were categorized by cytogenetic status according to National Comprehensive Cancer Network (NCCN) risk score^24^. The patient cohort comprised 18 patients with favourable risk, 23 with intermediate risk and 18 with poor risk score. A detailed list of cytogenetic abnormalities for patients with ‘AML with recurrent genetic abnormalities’ can be found online as Supplementary Table S1. Induction therapy was applied to 40 patients, the remaining patients (n = 22) were treated with other approved therapies (e.g. hydroxyurea) or best supportive care. Response to chemotherapy was defined according to European Leukemia Network (ELN) definition^25^. Complete response (CR) was defined as presentation with normocellular bone marrow containing <5% blasts and neutrophilic granulocytes in peripheral blood (PB) recovered to 1,500/μl and platelets to 100,000/μl. In contrast, complete remission with incomplete blood count recovery (CRi) lacked hematologic recovery in PB with neutrophil counts below 1,000/μl or platelets below 100,000/μl. CR after anthracycline-based induction therapy was reached in 65% of treated patients.

**Table 1:** Patients characteristics.

	Number of patients (%) (n = 62)
Sex	
Male	30 (48)
Female	32 (52)
Median age (years)	64 (range 21–86)
FAB classification	
M0	4 (6.5)
M1	15 (24)
M2	16 (26)
M3	4 (6.5)
M4	8 (13)
M5	6 (10)
M6	9 (14)
Unfavourable FAB	13 (20.5)
WHO classification	
AML with recurrent genetic abnormalities	29 (47)
AML with myelodysplasia-related changes	10 (16)
Therapy-related myeloid neoplasms	3 (5)
Myeloid neoplasms with germline predisposition	0 (0)
AML, not otherwise specified	20 (32)
Primary/secondary AML	
Primary	46 (74)
Secondary	16 (26)
Blood count	
WBC (G/L)	70 (range 10–448)
Hb (g/dl)	8.25 (range 3.8–12.9)
Plt (G/L)	42 (range 6–222)
NCCN risk score distribution	
Favourable	18 (29)
Intermediate	23 (37)
Poor	18 (29)
Not classified	3 (5)
Complete response after induction therapy^π^	26 (65)

FAB: French-American-British; WBC: white blood count; Hb: haemoglobin; plt: thrombocytes; NCCN: National Comprehensive Cancer Network; π only patients receiving anthracycline-based induction therapy, response assessment on day 25–35 after induction (CR, CRi).

### Prognostic evaluation of CD105 expression in AML

As a first step, expression of CD105 on the various cellular subsets within PBMCs of healthy donors (n = 8) was evaluated by flow cytometry. CD105 expression was not detectable on T cells (CD3^+^), B cells (CD19^+^) and natural killer (NK) (CD56^+^ CD3^−^) cells, while monocytes (CD14^+^) displayed substantial levels of CD105 (SFI mean 11.3) (Fig. S2A). This is in line with findings of other investigators reported for activated monocytes^26^.

**Figure 2:**
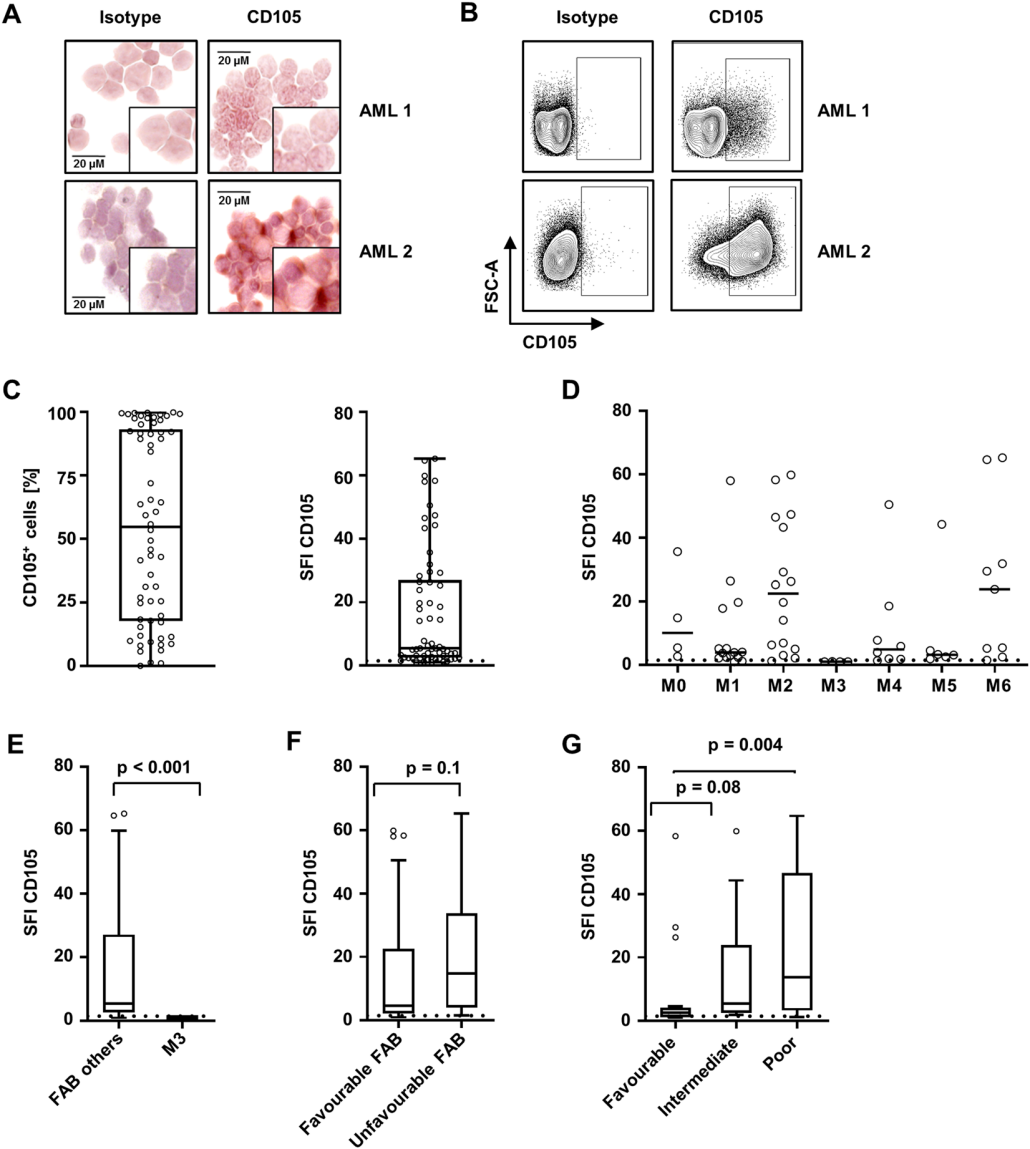
CD105 expression on malignant haematopoietic cells. (**A**) Immunohistochemistry of CD105 expression of two exemplary AML patients. (**B**) Exemplary gating for two AML samples with the respective isotype control and specific CD105 binding. Specific gating strategy and controls are shown in Fig. S2. (**C**–**G**) CD105 expression was analysed on AML cells by flow cytometry. SFI levels above 1.5 were considered as positive expression (dotted line) (**C**) CD105 expression on blasts of AML patients (n = 62) are depicted as percentage of CD105 positive blasts and SFI levels (boxplots with Tukey whiskers). (**D**–**F**) CD105 SFI levels are depicted for the different FAB types (single values, median; Kruskal-Wallis-test) (**D**), FAB M3 vs. others FAB (boxplots with Tukey whiskers; Mann-Whitney-test) (**E**) and according to favourable and unfavourable FAB classification (boxplots with Tukey whiskers; Mann-Whitney-test) (**F**) are shown. (**G**) Distribution of CD105 expression (SFI) throughout NCCN risk group (boxplots with Tukey whiskers; Kruskal-Wallis-test).

Next, leukaemic blasts of the AML patients were analysed for CD105 expression. To verify that our newly generated CD105 mAb is also valuable for immunohistochemistry, we stained some of the AML samples and could demonstrate comparable results regarding our flow cytometric analyses (Fig. 2A). The gating strategy for flow cytometric analyses with respective controls is exemplified with two AML samples depicted in Fig. 2B and Fig. S2. Highly variable surface levels ranging from complete lack of CD105 to SFI levels of 65.2 were detected, with 57 patients (92%) displaying relevant surface levels defined as SFI ≥ 1.5. Substantial variation was also observed for the percentage of CD105^+^ AML blasts within individual samples, ranging from 0% to nearly 100% positive leukaemic cells (Fig. 2C). Cases with monocytic differentiation (FAB M4 and M5) tended towards a surface expression of CD105 similar to the SFI levels observed with healthy monocytes (SFI mean 11.5 and 9.97, respectively). Cases with FAB M2 displayed the most heterogeneous CD105 expression profile (Fig. 2D), and a significant lower proportion of CD105 positive AML blasts was observed in patients with FAB M3 (SFI 1.06; 2.7%) compared to the other FAB types (Fig. 2E). Comparison of FAB types with favourable (M1, M2, M3, M4, M5) and unfavourable (M0, M6) prognosis^27–30^ revealed no significant difference, despite a trend to higher CD105 expression in the unfavourable FAB group (favourable vs. unfavourable, SFI mean 4.55 vs. 14.85; p = 0.1) (Fig. 2F).

Next, AML patients were grouped according to the National Comprehensive Cancer Network (NCCN) risk score. Interestingly, levels of CD105 expression correlated with NCCN risk: while the comparison of the favourable with the intermediate risk group revealed a strong tendency that did not reach statistical significance (SFI mean 8.28 vs. 14.2; p = 0.08), CD105 expression was significantly higher in the poor risk compared to the favourable NCCN risk group (SFI mean 24.18 vs. 8.28; p = 0.004) (Fig. 2G).

No significant correlation of CD105 was observed for age (>60 vs. <60 years), gender, WHO classification or primary vs. secondary AML as shown in Supplementary Table S2.

**Figure 3:**
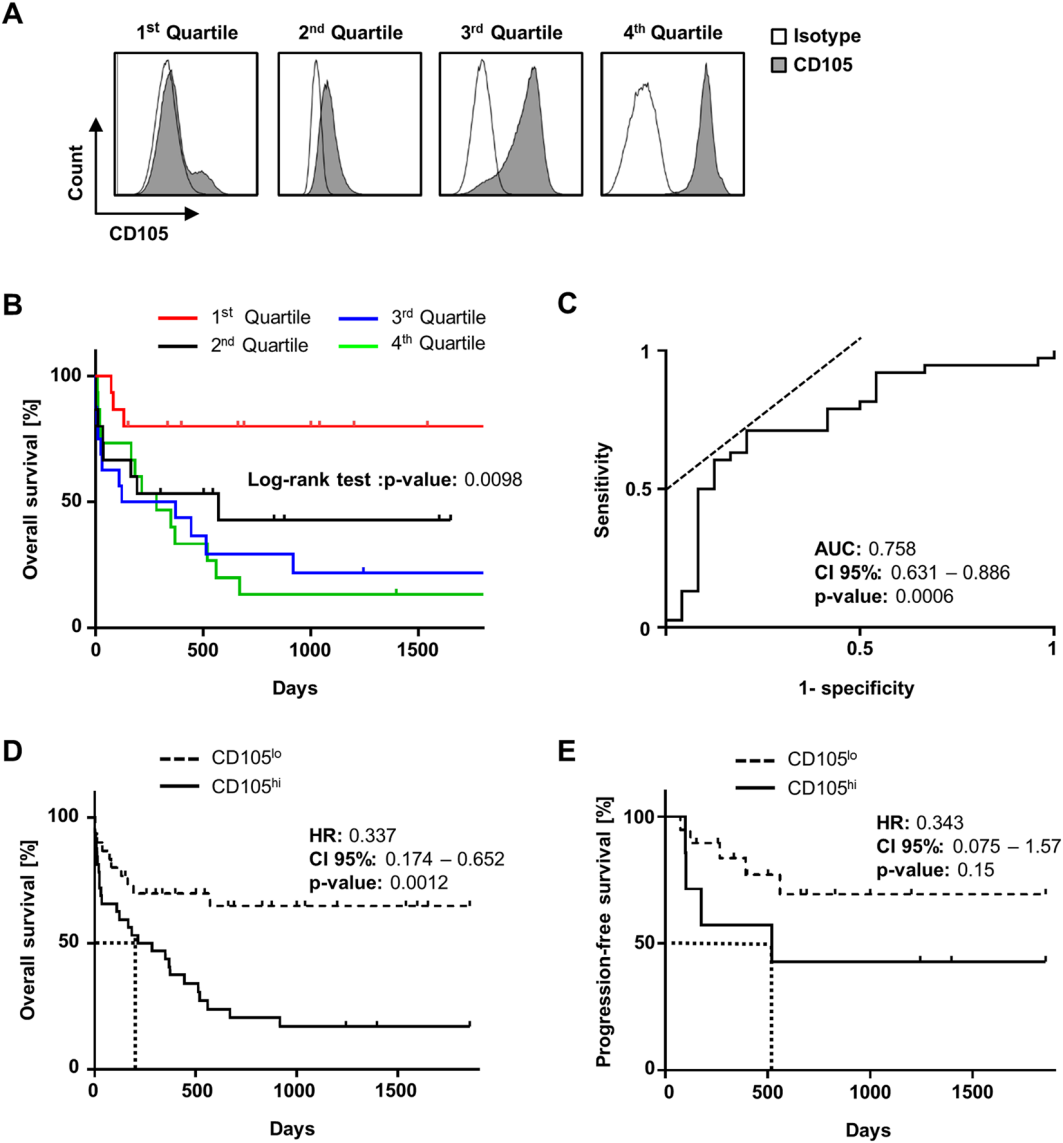
Impact of CD105 expression on clinical outcome. (**A**) Specific binding of the CD105 antibody (shaded peaks) and an isotype control (open peaks) at 5 nM on AML blasts from exemplary patients of quartiles 1–4 using flow cytometry. (**B**) Kaplan-Meier analysis of SFI levels of CD105. Overall survival (OS) of each SFI quartile (1^st^: 1–2.42; 2^nd^: 2.43–5.25; 3^rd^:5.26–26.37; 4^th^: 26.38–65.2) was plotted followed by statistical analysis with log-rank test. (**C**) ROC for SFI levels of CD105 and OS were plotted. The crossing of the dotted line and the ROC curve marks the Youden-index for the highest sensitivity and specificity. (**D**) Overall survival according to CD105^lo^ and CD105^hi^ expression in Kaplan-Meier analysis. Mean OS was reached in CD105^hi^ after 199 days (dotted line; log-rank test). (**E**) PFS after any AML specific treatment according to CD105^lo^ and CD105^hi^ expression in Kaplan-Meier analysis. In CD105^hi^ the mean PFS was 383 days (dotted line; log-rank test).

When patients were separated in quartiles (Q1-4) according to CD105 expression (Fig. 3A), a significant correlation of high surface levels with higher NCCN risk was observed (p = 0.019) (Table 2). Moreover, a significantly higher WBC was detected in the third quartile (Q3) compared to the others (mean 187.1 G/L; p < 0.01). WHO classification of AMLs revealed significantly more cases of acute myeloid leukaemia with myelodysplasia-related changes in the higher quartiles (0 vs. 3 vs. 4 vs. 3, respectively, p = 0.01). There was no difference between the quartiles with regard to age (p = 0.77), gender (p = 0.72) or favourable/unfavourable FAB classification (p = 0.17). Of note, while failing to achieve statistical significance (p = 0.06), a clear trend towards an inverse association of CD105 expression levels and achievement of CR after first anthracycline-based induction therapy was observed between the quartiles. To correlate CD105 expression in quartiles with OS, Kaplan-Meier analysis was performed and revealed that patients with low or absent CD105 expression (Q1) showed superior survival and did not reach median OS in the observation time, whereas for patients in the highest quartile (Q4) median OS was 284 days (p = 0.0098) (Fig. 3B).

**Table 2:** Distribution of patient characteristics according to CD105 quartiles.

	Number of patients (%)				p-value
	1^st^ quartile (CD105 SFI 1–2.42) n = 15	2^nd^ quartile (CD105 SFI 2.43–5.25) n = 16	3^rd^ quartile (CD105 SFI 5.26–26.37) n = 16	4^th^ quartile (CD105 SFI 26.38–65.2) n = 15	
Sex					
Male	7 (47)	10 (62)	7 (44)	8 (53)	0.72^‡^
Female	8 (53)	6 (38)	9 (56)	7 (47)	
Median age (years)	59 (range 29–81)	61 (range 21–80)	60 (range 21–86)	61 (range 38–81)	0.77^†^
FAB classification					0.014^‡^
M0	0 (0)	1 (6)	2 (12)	1 (7)	
M1	4 (27)	7 (44)	2 (12)	2 (13)	
M2	2 (13)	2 (13)	6 (38)	6 (40)	
M3	4 (27)	0 (0)	0 (0)	0 (0)	
M4	3 (20)	1 (6)	3 (19)	1 (7)	
M5	1 (6.5)	4 (25)	0 (0)	1 (7)	
M6	1 (6.5)	1 (6)	3 (19)	4 (26)	
Unfavourable FAB	1 (7)	2 (12)	5 (31)	5 (33)	0.17^‡^
WHO classification					0.01^‡^
AML with recurrent genetic abnormalities	12 (80)	6 (38)	3 (19)	8 (53)	
AML with myelodysplasia-related changes	0 (0)	3 (19)	4 (25)	3 (20)	
Therapy-related myeloid neoplasms	2 (13)	0 (0)	0 (0)	1 (7)	
Myeloid neoplasms with germline predisposition	0 (0)	0 (0)	0 (0)	0 (0)	
AML, not otherwise specified	1 (7)	7 (43)	9 (56)	3 (20)	
Primary/secondary AML					
Primary	12 (80)	10 (63)	13 (81)	11 (73)	0.61^‡^
Secondary	3 (20)	6 (37)	3 (19)	4 (27)	
Blood count					
WBC (G/L)	65.8	90.2	196.8	84.4	<0.01^†^
Hb (g/dl)	8.7	8.5	7.6	9	0.07^†^
Plt (G/L)	80	52	64	49	0.14^†^
NCCN risk score distribution					0.019^‡^
Favourable	9 (60)	6 (37)	1 (6)	2 (13)	
Intermediate	4 (27)	7 (44)	8 (50)	4 (27)	
Poor	2 (13)	3 (19)	5 (31)	8 (53)	
Not classified	0 (0)	0 (0)	2 (13)	1 (7)	
Complete response after induction therapy^π^	11 (84)	7 (78)	3 (33)	5 (56)	0.06^‡^

FAB: French-American-British; WBC: white blood count; Hb: haemoglobin; plt: thrombocytes; NCCN: National Comprehensive Cancer Network; π only patients receiving anthracycline-induction therapy, response assessment: on day 25–35 after induction (CR, CRi), Statistical analysis with ^‡^Pearson-Chi^2^ and ^†^students t-test.

These findings were confirmed by ROC curve analysis (AUC 0.76; 95% CI 0.63–0.89; p = 0.0006). To discriminate between patients with better or worse OS, a predictive cut-off value for CD105 surface expression was calculated by determination of the Youden Index and defined at an SFI value of 5.22 (sensitivity 71%; specificity 79%) (Fig. 3C). Then patients were accordingly grouped in a *CD105 low* (CD105^lo^, SFI < 5.22) and a *CD105 high* (CD105^hi^, SFI ≥ 5.22) cohort. A significant trend to an overrepresentation of unfavourable FAB types in CD105^hi^ AMLs was observed (3 vs. 10 patients; p = 0.04), while increased WBC was significantly associated with the CD105^hi^ cohort (mean WBC 78.4 vs. 142 G/L; p = 0.009). Notably, CR after anthracycline-based induction therapy occurred in a significantly higher number of cases in the CD105^lo^ than in CD105^hi^ group (84% vs. 39%; p = 0.01) which is in line with the observation that the CD105^hi^ group comprised more NCCN poor risk patients (17% vs. 43%, p = 0.0019). No significant difference was observed for age, gender or primary/secondary AML (Table 3).

**Table 3:** Distribution of patient characteristics according to CD105^lo^ and CD105^hi^.

	Number of patients (%)		p-value
	CD105^lo^ (SFI < 5.22) n = 30	CD105^hi^ (SFI ≥ 5.22) n = 32	
Sex			
Male	17 (55)	15 (48)	0.80^┘^
Female	14 (45)	16 (52)	
Median age (years)	63 (range 21–81)	67 (range 21–86)	0.87^†^
FAB classification			0.013^‡^
M0	1 (4)	3 (9)	
M1	10 (33)	5 (15)	
M2	4 (13)	12 (37)	
M3	4 (13)	0 (0)	
M4	4 (13)	4 (13)	
M5	5 (17)	1 (3)	
M6	2 (7)	7 (23)	
Unfavourable FAB	3 (10)	10 (32)	0.04^┘^
WHO classification			0.14^‡^
AML with recurrent genetic abnormalities	18 (60)	11 (34)	
AML with myelodysplasia-related changes	3 (10)	7 (22)	
Therapy-related myeloid neoplasms	2 (7)	1 (3)	
Myeloid neoplasms with germline predisposition	0 (0)	0 (0)	
AML, not otherwise specified	7 (23)	13 (41)	
Primary/secondary AML			
Primary	22 (73)	24 (75)	0.88^┘^
Secondary	8 (27)	8 (25)	
Blood count			
WBC (G/L)	79.4	139.5	0.0086^†^
Hb (g/dl)	8.6	8.3	0.73^†^
Plt (G/L)	58	47	0.74^†^
NCCN risk score distribution			0.0019^‡^
Favourable	15 (50)	3 (10)	
Intermediate	10 (33)	13 (40)	
Poor	5 (17)	13 (40)	
Not classified	0 (0)	3 (10)	
Complete response after induction therapy^π^	18 (82)	8 (44)	0.014^┘^

FAB: French-American-British; WBC: white blood count; Hb: hemoglobin; Plt: thrombocytes; NCCN: National Comprehensive Cancer Network; π only patients receiving anthracycline-based induction therapy, response assessment: on day 25–35 after induction (CR, CRi). Statistical analysis with ^┘^Fisher’s exact-test, ^‡^Pearson-Chi^2^ and ^†^Students t-test.

Finally, the predictive value of the defined cut-off for CD105 expression was determined using Kaplan-Meier analyses, which confirmed a significant advantage in OS for the CD105^lo^ patients (hazard ratio (HR) 0.34; 95% CI 0.17–0.66, p = 0.0012) (Fig. 3D). This difference almost held true in analyses of progression free survival (PFS) after response to anthracycline based induction therapy, but failed to reach statistical significance (HR 0.34; 95% CI 0.08–1.6, p = 0.15) (Fig. 3E). To confirm these results, multivariate analysis including age (<60 vs. ≥60 years), WBC, primary/secondary AML, risk profile according NCCN and CD105 expression was conducted. A HR of 0.25 was calculated for CD105 (p = 0.0044), with a slightly better HR of 0.23 reached for age (<60 vs. ≥60 years, p = 0.0008); no other variable showed significant impact on OS. Multivariate analysis for survival can be found as Supplementary Table S3.

## Discussion

The TGF-beta co-receptor CD105 plays a major role in foetal, adult and malignant angiogenesis. Previous studies reported that CD105 expression on tumour vessels of endometrial, colorectal, breast, prostate, and non-small cell lung cancer^15–20^ correlates with poor outcome. Despite the fact that CD105 reportedly is expressed on malignant cells in various haematopoietic malignancies, data on its prognostic relevance in leukaemia are still not available. In this study we demonstrate that CD105 expression on leukaemic blasts strongly correlates with poor OS and PFS, poor risk profile according to NCCN and decreased response to anthracycline-based induction therapy. On the contrary, patients with absent or low CD105 levels on AML blasts revealed the best OS. We established a cut-off value of 5.22 as calculated by receiver-operating characteristics to define CD105 as marker for risk stratification in AML.

Substantial expression of CD105 was observed by flow cytometry in 57 (92%) of the total of 62 analysed patients in our cohort. Notably, in a previous study, where 119 bone marrow samples from AML patients were analysed by immunohistochemistry, CD105 positivity as defined by staining of at least 1/5 of all myeloblasts was reported in 24% of cases^22^. In another study, flow cytometric analysis of CD105 expression in 336 cases of acute lymphoblastic leukaemia (ALL) and 666 bone marrow samples from AML patients revealed CD105 expression in 68.4% and 37% of cases, respectively^21^. However, no clear cut-off value was defined by the authors to differentiate between cases with good and bad prognosis. In the third study so far published on the expression of CD105 in haematopoietic malignancies, Della Porta *et al*. employed flow cytometric analysis of bone marrow specimens and observed CD105 expression on all erythroblasts from patients with MDS^23^. In our study, CD105 expression >SFI 1.5, the threshold defined by us for positivity, was observed in 92% of the analysed cases. CD105 was not detectable in any of the analysed cases of promyelocytic leukaemia (FAB M3). The same was observed by Cosimato and co-workers^21^, whereas Chakhachiro *et al*. described CD105 expression on promyelocytic blasts when using immunohistochemistry^22^. The discrepancy might be due to the fact that flow cytometry, like employed in the work of Cosimato and us, might be more specific and in contrast to immunohistochemistry only detects surface expressed CD105. In addition, we employed a novel CD105 antibody which was particularly selected for its sensitivity in flow cytometric staining, but applicable for immunohistochemistry as well. A direct comparison of k-ro23 with two commercially available antibodies (clones SN6, 166707) showed a higher affinity of our antibody as reflected by saturation at lower concentrations. However, the maximum signal was lower compared to 166707, the clone used by Cosimato *et al*. and comparable with signal intensities of SN6.

Furthermore, in contrast to the above mentioned publication, we defined a clear cut-off to differentiate between positive and negative cases. While the discrepancy could also be due to inter-individual differences between the patients or the material used for analysis (bone marrow versus peripheral blood), the fact that our antibody was particularly developed to this end may explain the higher rate of positive cases in our study.

CD105 was shown to induce activation and proliferation of endothelial cells by promotion of TGF-beta/ALK-1 signalling and direct inhibition of TGF-beta/ALK-5 signalling^8,31,32^. It interacts with TGF-beta receptor I and TGF-beta receptor III as well as activating bone morphogenetic protein (BMP)-2^7^. CD105 signalling has also been shown to drive angiogenesis^11^, and its expression is upregulated by hypoxic endothelium via hypoxia inducible factor (HIF) 1 alpha, which inhibits apoptosis of endothelial cells^33,34^. Notably, it has recently been reported that upregulation of HIF 1 alpha on AML blasts is correlated with the presence of multi-drug-resistance transporters such as multi-drug-resistance protein (MDR) 1 and MDR associated protein (MRP) 1^35^. While the functional role of CD105 that underlies the association with inferior OS observed in our study remains unclear, it is tempting to speculate that CD105 might influence the occurrence of MDR and thereby reduce the response to chemotherapy.

Moreover, CD105 has been described as important mediator of stem cell properties in myeloid precursor cells. CD105^+^CD34^+^ expressing cells comprise a subgroup of long-term repopulating haematopoietic stem cells (HSCs) which are crucial for maintaining the HSC pool^36,37^. Within this population, CD105 seems to be necessary for re-entry into the quiescence state, since its deletion led to engraftment failure and reduced numbers of HSCs in G0 phase in a xenograft model^38^. In line, CD105^+^ AML blasts showed faster bone marrow engraftment than CD105^−^ blasts and led to decreased survival in a xenograft mouse model. This effect could be prevented by application of a blocking CD105 antibody, thus suggesting a critical role of CD105 in disease progression of AML^39^. Together, these data provide another potential explanation for the observed effect in our study and identify AML as potential disease entity that might benefit from CD105 targeted therapeutics.

Overall, the data presented in our study support the value of immunophenotyping as a fast and reliable method to achieve information on prognosis in AML in general and identify CD105 as a novel prognostic marker for adverse disease course in particular. Notably, a CD105 therapeutic antibody (TRC105) is already available and was well tolerated upon phase 1 and 2 clinical evaluation in patients with soft tissue sarcoma, hepatocellular and urothelial carcinoma^40–42^. In combination with established treatments, TRC105 achieved overall response rate of 25% in hepatocellular carcinoma and prolonged PFS in patients with soft tissue sarcoma^41,42^. While CD105 has so far not been analysed as therapeutic target in AML patients, our data point to the fact that this might be a promising approach. However, certainly additional analyses in preclinical models of AML including sophisticated mouse models that enable a better understanding of the functional role of CD105 are required to provide a broader basis for potential future clinical studies that might ultimately improve treatment of AML.

## Methods

### Patient samples

Peripheral blood samples of 62 patients with AML at primary diagnosis were collected. All experiments were carried out in accordance with the Helsinki protocol and the Ethics Committee of the University of Tübingen vote (13/2007V) between 2006 and 2017. All experimental protocols were approved by the Ethics Committee of the University of Tübingen (13/2007V). Informed consent was obtained from all patients and healthy donors and – in case of subjects younger than 18 years – from a parent or legal guardian.

Peripheral blood mononuclear cells (PBMC) of patients were isolated by density gradient centrifugation und used for flow cytometric analysis. Median observational time for all patients was 360 days (95% CI 166–545 days). Diagnosis and classification of AML samples was based on morphology and cytochemistry of bone marrow according to the French-American-British (FAB) classification^43,44^. Cytogenetic analyses were performed with standard methods at the University of Ulm.

### Generation, purification and characterization of a CD105 antibody

The murine monoclonal anti-CD105 antibody (clone K-ro23) was generated by immunization of female BALB/c mice (Charles River, Wilmington, MA) with gamma-irradiated Sp2/0-Ag14 cells (ATCC, Manassas, VA) transfected with human CD105. Immunization was performed intraperitoneally together with CpG oligonucleotides to enhance immunogenicity. Spleen cells were then fused with Sp2/0-AG14 cells according to the protocol established by Kohler and Milstein^45^. Supernatants of the resulting hybridoma cells were screened by flow cytometric analysis using CD105^+^ NALM-16 (DMSZ, Braunschweig, Germany) and CD105-transfected Sp2/0 cells, while mock transfected Sp2/0 cells served as negative control. CD105 antibody was purified using protein A affinity chromatography.

For SDS-PAGE analysis, 3 µg of protein was diluted 1:1 with Laemmli sample buffer (Bio-Rad, Hercules, CA) with or without beta-mercaptoethanol (SERVA Electrophoresis, Heidelberg, Germany) as reducing agent. Samples were analysed on a 10% Mini-PROTEAN ® TGX™ Precast Protein Gel (Bio-Rad). Gels were stained with Roti®-Blue colloidal coomassie blue staining solution (Carl Roth, Karlsruhe, Germany). Antibody purity was confirmed by analytical size exclusion chromatography with a 200 Increase 10/300GL column (GE Healthcare, Chicago, IL).

### Flow cytometry

PBMC of AML patients and healthy donors as well as different leukaemic and solid tumour cell lines (purchased from DSMZ) were incubated with human IgG (Sigma-Aldrich, St. Louis, MO) solution prior to staining and then washed. Unconjugated anti-CD105 mAb (clone K-ro23 and clone SN6, Santa Cruz Biotechnology, Dallas, TX) or isotype control (clone MPC-11, BD Biosciences, Heidelberg, Germany) as well as the comparative anti-CD105 clone 166707 (R&D Systems, Minneapolis, MN) and the respective isotype control (IgG1 isotype, clone MG1-45, BioLegend) were added at 5 nM, followed by a mouse-specific PE conjugate (1:100). AML cells were identified according to the immunophenotype obtained at diagnosis by staining for CD33, CD34, CD38 and/or CD117, and dead cells were excluded based on 7-AAD (BioLegend) positivity. Fluorescence-conjugates (CD33, CD34, CD38 and CD117, all from BioLegend) were used in 1:100–1:200 dilutions. Measurements were conducted using a FACSCanto II or a LSR Fortessa (BD Biosciences) and data analysis was performed using FlowJo_V10 software (FlowJo LCC, Ashland, OR). Specific fluorescence indices (SFIs) were calculated by dividing median fluorescence obtained with anti-CD105 mAb by median fluorescence obtained with the isotype control. Expression was considered positive in case of SFI ≥ 1.5.

### Cytospins and immunohistochemistry

Cytospins of ~200.000 AML cells in PBS with 1% BSA were prepared and cells were fixed with methanol for 10 minutes at −20 °C. For immunohistochemistry, cytospins were Fc-blocked with PBS containing 1% BSA + hIgG (10 µg/mL) and peroxidases were inactivated with 3% H_2_O_2_ +1% NaN_3_ followed by Biotin blocking system (DAKO Agilent technologies, Santa Clara, CA). Incubation with anti-CD105 (K-ro23) or respective isotype control antibodies (10 µg/mL) was performed for 2 h followed by a rabbit anti-mouse biotin F(ab‘)_2_ conjugate (1:200) (DAKO). After incubation with streptavidin/HRP (DAKO) (1:1000) staining was visualized by 10–30 sec exposition with DAB Chromogen system (DAKO) and subsequently counterstained with haematoxylin. Prior to an ascending ethanol preservation and Xylol treatment, cytospins were covered with Neo-Mount mounting medium (Merck Millipore, Darmstadt, Germany).

### Statistical analysis

Data are displayed as mean +/− SEM, boxplot as mean with 25% or 75% quantiles and Tukey or min/max whiskers. The 2-tailed unpaired students-t test, Mann-Whitney-/Kruskal-Wallis-test, one-way ANOVA or Fisher´s exact test were used to compare individual groups. Distribution of overall survival (OS) was calculated by the Kaplan-Meier method. Log-rank test was performed to test the difference of survival between groups. For predictive cut-off value estimation, we sub-grouped CD105 SFI with respect to corresponding OS times. Receiver-operating-characteristics (ROC) analysis was performed using JMP® Pro and value of highest Youden index was used as cut-off. Cut‐off values enabled further separation of cases with better or worse prognosis, as shown e.g., in Kaplan–Meier analysis. Statistical analyses were conducted using GraphPad Prism 8.1.0 and JMP® Pro (SAS Institute Inc., Version 14.2) software. P values of <0.05 were considered statistically significant.

## Supplementary information

Supplementary Information

## Data Availability

The corresponding author had full access to all the data in the study and all authors shared final responsibility for the decision to submit for publication.
