# Supplementary material for: CD105 (Endoglin) as negative prognostic factor in AML
Source: Sci Rep. 2019 Dec 4;9:18337. doi: 10.1038/s41598-019-54767-x (PMC6892812; doi:10.1038/s41598-019-54767-x)
Supplement: Supplementary file 1 — Supplementary Information [file 41598_2019_54767_MOESM1_ESM.pdf]

Supplementary Information

Article in *Scientific Reports*

CD105 (Endoglin) as negative prognostic factor in AML

Joseph Kauer, Karolin Schwartz, Claudia Tandler, Clemens Hinterleitner, Malte Roerden, Gundram Jung, Helmut R. Salih, Jonas S. Heitmann, Melanie Märklin

Supplementary Table S1. Cytogenetic abnormalities for patients with 'AML with recurrent genetic abnormalities' according to WHO classification

Classification of 'AML with recurrent genetic abnormalities'	Number of patients (n = 29)
AML with t(8;21)(q22;q22.1); *RUNX1-RUNX1T1*	0
AML with inv(16)(p13.1q22) or t(16;16)(p13.1;q22); *CBFB-MYH11*	1
APL with PML-*RARA*	4
AML with t(9;11)(p21.3q23.3); *MLLT3-KMT2A*	3
AML with t(6;9)(p23;q34.1); *DEK-NUP214*	2
AML with inv(3)(q21.3;q26.2) or t(3;3)(q21.3;q26.2); *GATA2, MECOM (EVI1)*	0
AML (megakaryoblastic) with t(1;22)(p13.3;q13.3); *RBM15-MKL1*	0
AML with *BCR-ABL1*	0
AML with mutated *NPM1*	15
AML with biallelic mutations of *CEBPA*	4
AML with mutated *RUNX1*	0

Supplementary Information

Supplementary Table S2. Distribution of patients characteristics according to mean CD105 expression

	Mean SFI CD105 Expression (+/- SEM) (n = 62)	p-value
Sex		0.91
Male	16.1 (3.7)	
Female	16.0 (3.0)	
Age (years)		0.64
> 60 years	17.8 (4.1)	
< 60 years	14.7 (3)	
WHO classification		n.s.
AML with recurrent genetic abnormalities	17.2 (4.2)	
AML with myelodysplasia-related changes	18.9 (6.3)	
Therapy-related myeloid neoplasms	12.8 (11.4)	
Myeloid neoplasms with germline predisposition	n.d.	
AML, not otherwise specified	14.0 (3.1)	
Primary/secondary AML		0.83
Primary	16.8 (2.9)	
Secondary	13.9 (4.6)	

n.s. = not significant; n.d. = not done.

Supplementary Information

Supplementary Table S3. Multivariate analysis for survival

	n	HR	95-%-CI	p-value
CD105 expression				
CD105^{lo}	31			
CD105^{hi} ±	31	0.25	0.09–0.64	0.0044
Age				
< 60 years	27			
> 60 years±	35	0.23	0.09-0.54	0.0008
NCCN risk				
Favourable±	18			
Intermediate	23	1.56	0.51-4.81	0.43
Poor	18	1	0.32-3.11	0.99
Not classified	3			
Primary vs. secondary AML				
Primary	46			
Secondary±	16	0.94	0.43-2.03	0.87
FAB classification				
Favourable risk	49			
Unfavourable risk±	13	1.7	0.72-4.05	0.23
Blood count				
WBC	62	1.99	0.42-7.95	0.36

NCCN: National Comprehensive Cancer Network; FAB: French-American-British; WBC: white blood count; ±reference group.

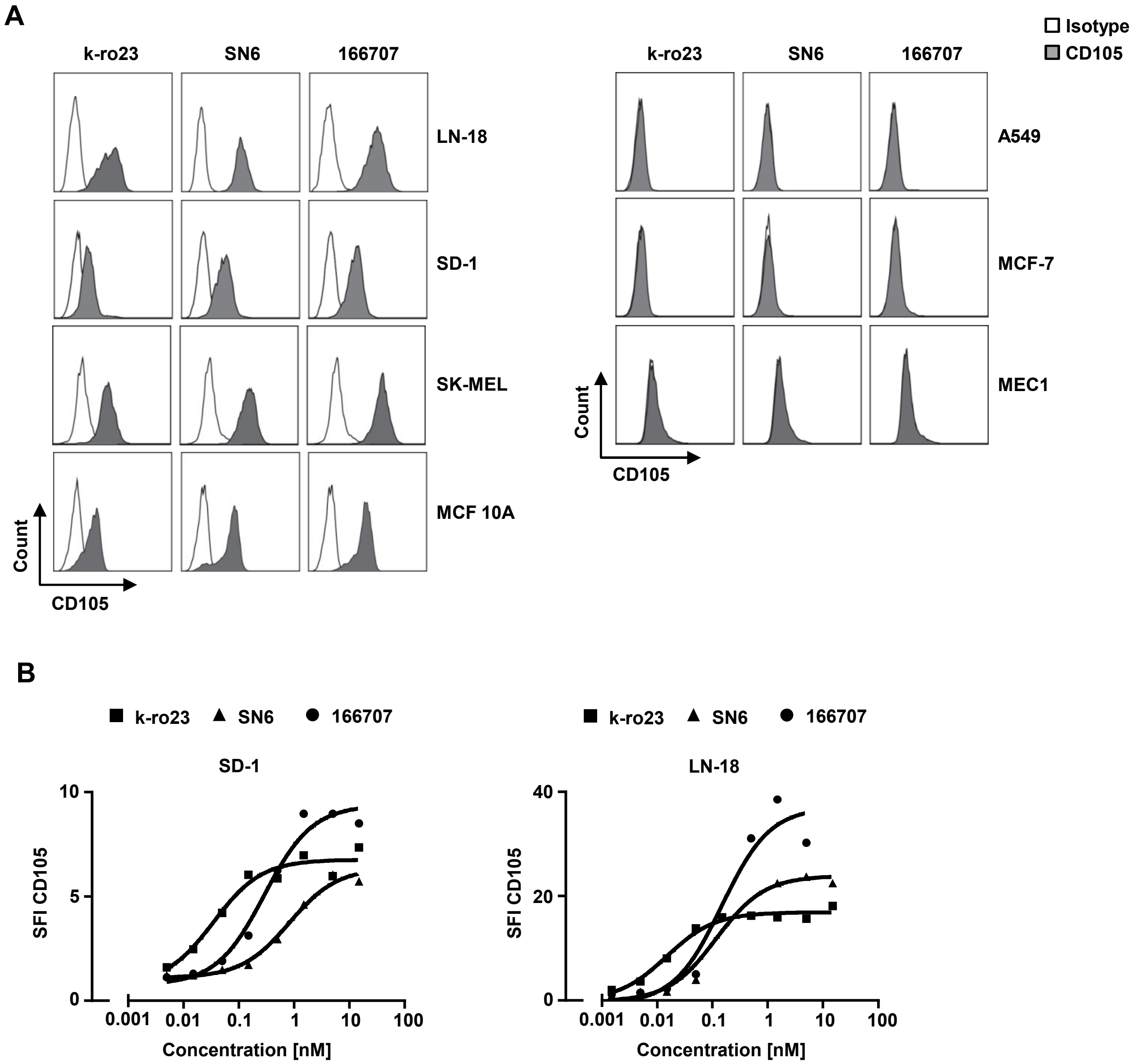

Supplementary Figure S1: CD105 expression on different cell lines

CD105 expression was analyzed by flow cytometry staining of different cell lines. **A** Specific binding of the CD105 antibodies k-ro23, SN6 and 166707 (shaded peaks) and the respective isotype controls (open peaks) at 5nM. Cell lines with endogenous CD105 expression and CD105^{neg} cell lines were used as indicated. **B** Titration of the different CD105 clones on SD-1 and LN-18 cells. Specific fluorescence intensity (SFI) levels are shown.

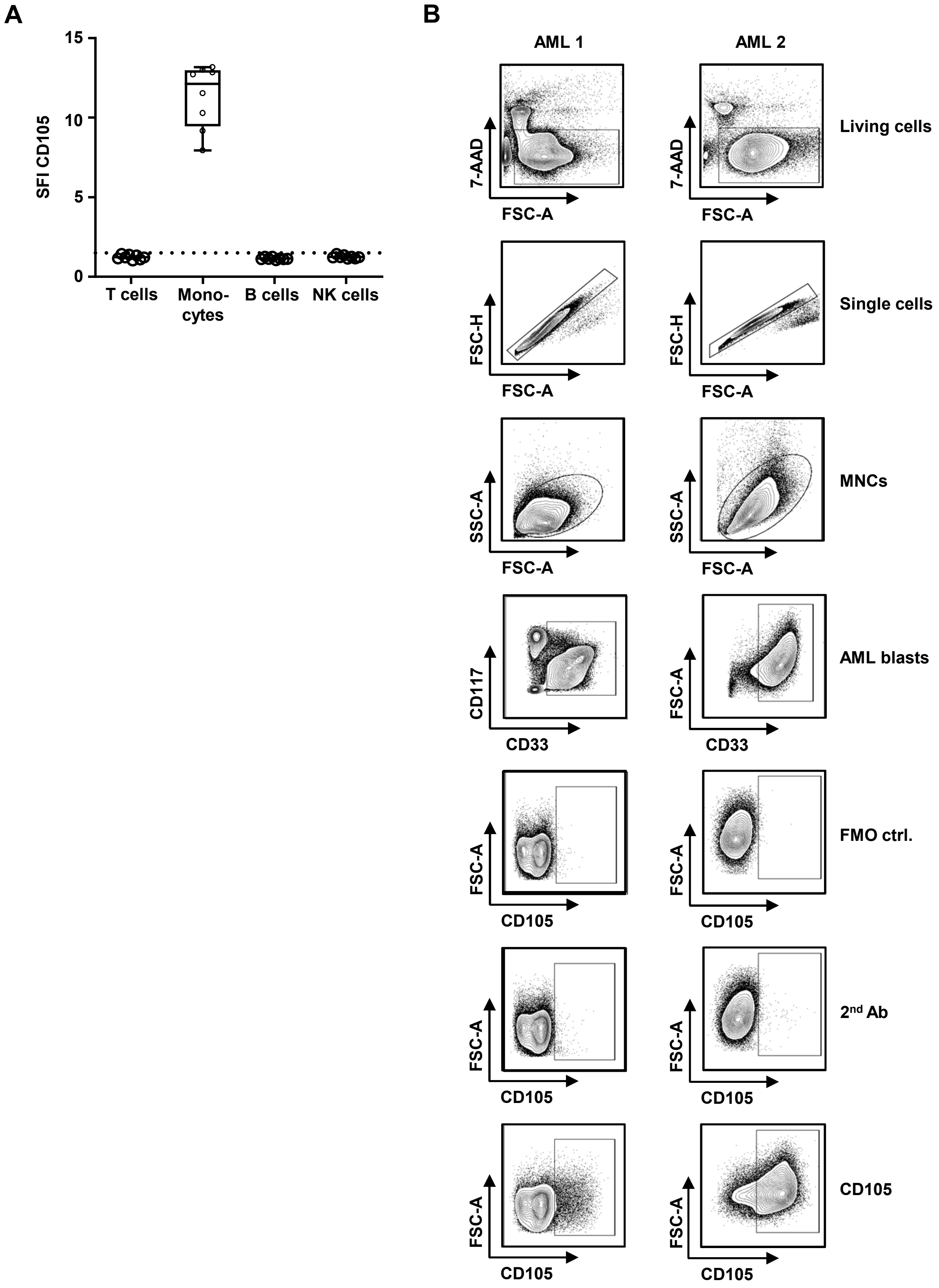

Supplementary Figure S2: Analyses of CD105 expression on healthy and malignant hematopoietic cells

(**A**) CD105 expression was analysed on B ($CD19^+$), NK ($CD56^+ CD3^-$), T cells ($CD3^+$) and monocytes ($CD14^+$) from eight healthy donors by flow cytometry. SFI levels above 1.5 were considered as positive expression (dotted line). (**B**) Gating strategy for two exemplary AML samples: Living cells (7AAD-), single cells, mononuclear cells, blast marker (AML1: CD33/CD117, AML2: CD33/FSC-A) and CD105 expression with the respective fluorescence-minus-one (FMO) and the 2nd mAb only control.
